# Heterozygosity for ADP-ribosylation factor 6 suppresses the burden and severity of atherosclerosis

**DOI:** 10.1371/journal.pone.0285253

**Published:** 2023-05-10

**Authors:** Venkateswara R. Gogulamudi, Md Torikul Islam, Jessica R. Durrant, Adelola O. Adeyemo, Daniel W. Trott, Mi Ho Hyuhn, Weiquan Zhu, Anthony J. Donato, Ashley E. Walker, Lisa A. Lesniewski

**Affiliations:** 1 Department of Internal Medicine, Division of Geriatrics, The University of Utah, Salt Lake City, Utah, United States of America; 2 Department of Nutrition and Integrative Physiology, The University of Utah, Salt Lake City, Utah, United States of America; 3 Dallas Tissue Research, Farmers Branch, Texas, Dallas, United States of America; 4 Department of Internal Medicine, Division of Cardiovascular Medicine, The University of Utah, Salt Lake City, Utah, United States of America; 5 Department of Pathology, The University of Utah, Salt Lake City, Utah, United States of America; 6 Program of Molecular Medicine, The University of Utah, Salt Lake City, Utah, United States of America; 7 Geriatric Research Education and Clinical Center, Veteran’s Affairs Medical Center-Salt Lake City, Salt Lake City, Utah, United States of America; 8 Department of Biochemistry, The University of Utah, Salt Lake City, Utah, United States of America; 9 Nora Eccles Harrison Cardiovascular Research and Training Institute, The University of Utah, Salt Lake City, Utah, United States of America; 10 Department of Human Physiology, The University of Oregon, Eugene, Oregon, United States of America; Medical College of Georgia at Augusta University, UNITED STATES

## Abstract

Atherosclerosis is the root cause of major cardiovascular diseases (CVD) such as myocardial infarction and stroke. ADP-ribosylation factor 6 (Arf6) is a ubiquitously expressed GTPase known to be involved in inflammation, vascular permeability and is sensitive to changes in shear stress. Here, using atheroprone, *ApoE*^*-/-*^ mice, with a single allele deletion of *Arf6* (HET) or wildtype *Arf6* (WT), we demonstrate that reduction in Arf6 attenuates atherosclerotic plaque burden and severity. We found that plaque burden in the descending aorta was lower in HET compared to WT mice (p˂0.001) after the consumption of an atherogenic Paigen diet for 5 weeks. Likewise, luminal occlusion, necrotic core size, plaque grade, elastic lamina breaks, and matrix deposition were lower in the aortic root atheromas of HET compared to WT mice (all p≤0.05). We also induced advanced human-like complex atherosclerotic plaque in the left carotid artery using partial carotid ligation surgery and found that atheroma area, plaque grade, intimal necrosis, intraplaque hemorrhage, thrombosis, and calcification were lower in HET compared to WT mice (all p≤0.04). Our findings suggest that the atheroprotection afforded by *Arf6* heterozygosity may result from reduced immune cell migration (all p≤0.005) as well as endothelial and vascular smooth muscle cell proliferation (both p≤0.001) but independent of changes in circulating lipids (all p≥0.40). These findings demonstrate a critical role for Arf6 in the development and severity of atherosclerosis and suggest that Arf6 inhibition can be explored as a novel therapeutic strategy for the treatment of atherosclerotic CVD.

## Introduction

Atherosclerosis is an inflammatory arterial disease that is characterized by the deposition of fatty streaks in the lumen of arteries [1–3]. The severity of this disease is determined not only by the size but also by the nature (e.g., composition and stability) of the plaque [2]. While large plaques may lead to arterial occlusion and cardiovascular events; more commonly, plaque instability leads to plaque rupture or thrombotic events leading to myocardial infarction, stroke, and the associated morbidity and mortality [2]. Although, numerous hemodynamic and circulating factors contribute to disease progression, dyslipidemia, particularly elevated blood low-density lipoprotein (LDL), is a direct cause of atherosclerosis [1,2,4,5]. Much of the previous efforts targeting atherosclerotic diseases were focused on lowering cholesterol, resulting in the emergence of cholesterol lowering drugs such as statins [6,7]. However, despite the availability and widespread use of statins and successful management of blood cholesterols, atherosclerotic cardiovascular diseases remain the leading cause of global death [7,8]. Therefore, elucidating novel mechanisms and molecular mediators that regulate atherosclerotic plaque burden would have significant clinical impacts.

Arterial regions that are exposed to oscillatory shear stress, such as branch points and curvatures, are particularly susceptible to atherosclerosis [2]. Because of their spatial location on the luminal surface of arteries, endothelial cells are the first responders to proatherogenic shear stress related stimuli [3,4]. Exposure to oscillatory shear stress activates endothelial cells resulting in impairments in barrier function and promotion of LDL infiltration and oxidation in the subintimal space [2,4]. Oxidized LDL triggers immune cell migration and foam cell formation which subsequently results in vascular smooth muscle cell and endothelial cell proliferation and migration into the lumen area [9–11]. Thus, a coordinated action of endothelial cells, immune cells and vascular smooth muscle cells leads to the development of atheroma [12,13]. Continued cellular proliferation, inflammation, and hypoxia in the growing plaque contribute to further luminal occlusion and the formation of a necrotic core covered by a fibrous cap, the rupture of which can lead to thrombotic events and infarction in advanced disease [2]. Therefore, identification of molecular mediators and signaling pathways that regulate shear sensing, cell migration and proliferation in atherosclerotic diseases may lead to novel therapies.

ADP-ribosylation factor 6 (Arf6) is a small GTPase that interacts with the Rac signaling pathway [14–16]. It is a ubiquitously expressed protein that is best described for its role in trafficking events, such as endocytic recycling and cytoskeletal remodeling [14]. In the endothelium, Arf6 plays a critical role in controlling barrier function and inflammation [15,17]. Arf6 also plays a role in angiogenesis, cell migration [14,16], sensing shear patterns, and cytoskeleton remodeling [18]. As many of these processes impacted by Arf6 signaling are known to play important roles in the development of atherosclerotic lesions; here, we sought to determine if Arf6 could modulate atherosclerotic plaque burden, and severity. We hypothesized that genetically reducing Arf6 would attenuate atherosclerotic plaque accumulation, reduce atheroma size, and improve atheroma quality. To test this hypothesis, we examined the burden and severity of atherosclerosis using *Arf6* heterozygous (HET) and wildtype (WT) littermate control mice on the apolipoprotein E knockout (*ApoE*^*-/-*^*)* background. *ApoE*^*-/-*^ mice develop atherosclerotic plaques in aortic regions with oscillatory sheer even when fed a normal chow diet [19,20]. The development and severity of these plaques can be accelerated by consumption of an atherogenic diet. However, mouse atherosclerotic plaques are spontaneous and simple in nature, while humans develop advanced and complex lesions. To overcome this limitation, we performed partial carotid ligation (PCL) of the left carotid artery [19,21,22]. PCL is a surgical model of induced, acute, oscillatory sheer that, when combined with consumption of an atherogenic diet in *ApoE*^*-/-*^ mice, leads to the development of advanced atherosclerotic lesions within 2–5 weeks [21].

## Materials and methods

### Animals

Apolipoprotein E knockout (*ApoE*^*-/-*^) mice obtained from Jackson Laboratory were crossed with *Arf6*^*+/-*^ *mice to produce atheroprone*, wildtype (WT: *Arf6*^*+/+*^*ApoE*^*-/-*^) or heterozygous (HET: *Arf6*^*+/-*^*ApoE*^*-/-*^). We obtained the *Arf6*^*+/-*^ strain from Drs. Dean Li and Weiquan Zhu at the University of Utah, who developed and previously described this strain [15]. Male and female, WT and HET littermates were studied at 4–7 mo of age. All animal procedures conform to the Guide for the Care and Use of Laboratory Animals [23] and were approved by the Institution Animal Care and Use Committees at the University of Utah and Veteran’s Affairs Medical Center-Salt Lake City (VAMC-SLC) [24].

### Arf6 expression

To determine if HET mice demonstrate reduced *Arf6* gene expression, endothelial and smooth muscle cells enriched carotid artery fractions were collected from WT and HET mice. Endothelial cell enriched fractions were obtained by flushing excised carotid arteries with 100 μL of trizol. The effluent was collected and RNA isolated. To assess *Arf6* gene expression in a smooth muscle enriched fraction, the remainder of the artery was mechanically disrupted, and RNA was isolated using RNeasy Mini Kit (Qiagen) as previously described [25–27] and according to the manufacturer’s protocol. mRNA was converted into cDNA using QuantiTect Reverse Transcription Kit (Qiagen) according to the manufacturer’s protocol. Gene expression for Arf6 was assessed by quantitative PCR on 96-well plates using SsoFast EvaGreen Supermixes (Bio-Rad) with the Bio-Rad CFX™ Real-Time System. Gene expression was normalized to 18s and the fold change was calculated using 2^-ΔΔCt^ method. Primer sequences were as follows: *18s* F 5′-TAGAGGGACAAGTGGCGTTC-3′, *18s* R 5′- CGCTGAGCCAGTCAGTGT-3′; *Arf6* F 5′-ATGGGGAAGGTGCTATCCAAAATC-3′, *Arf6* R 5′-GCAGTCCACTACGAAGATGAGACC-3′. In addition, endothelial cells were isolated from the lungs of WT and HET mice (please see the cell proliferation section for details), lysed and protein isolated for assessment of Arf6 expression by standard Western blot techniques using anti-mouse primary antibodies against Arf6 (rabbit monoclonal; 1:1000; 20kDa; Cell Signaling), β-actin (mouse monoclonal; 1:1000; 40kDa; Abcam). Goat anti-rabbit and anti-mouse IgG (H+L)-HRP conjugate (Bio-Rad) was used as the secondary antibodies.

### Evaluation of plaque burden in descending aorta

To assess the impact of modulating Arf6 expression on the burden of lipogenic aortic plaques, tissues were collected from WT and HET mice 5 weeks after initiation of an atherogenic Paigen diet (Teklad Custom Diet, TD.88051, 15.8% fat, 1.25% cholesterol and 0.5% sodium cholate). The descending aorta was dissected and stained with Sudan IV to assess plaque area. The remaining heart with attached aortic root and carotid arteries were prepared for histological analyses. Plaque area was measured using ImageJ software (NIH) and normalized to total aortic area and percent plaque area was used in the analyses.

### Evaluation of aortic root lesion size and quality

To evaluate the aortic root, the heart was embedded in paraffin and sectioned at ~4 μm from the base of the heart into the aortic valve and root. Aortic root slides were collected when a complete tunica media was visible. Slides were stained with hematoxylin and eosin (H&E), Masson’s trichrome (MT) or Movat’s pentachrome stain [26,28]. For each staining type, we analyzed one slide per aortic root. All slides were evaluated by a blinded ACVP-board certified veterinary pathologist, Dr. Jessica Durrant. Plaque morphology grade was assigned based on human medical classification (1 = intimal thickening, 2 = intimal xanthoma, 3 = pathological intimal thickening, 3.5 = intimal thickening with erosion, 4 = fibrous cap atheroma, 4.5 = fibrous cap atheroma with erosion, 5 = thin fibrous cap atheroma, 5.5 plaque rupture, 6 = calcified nodule, 7 = fibrocalcific plaque) [29]. Using ImageJ software, measurements were performed on Movat’s pentachrome-stained atheromas: atheroma and necrotic core area, and minimum and maximum fibrous cap thickness when appropriate (i.e., fibrous cap measures only for plaque grades 4 and 5), and percent luminal occlusion was calculated ([area internal to the internal elastic lamina—luminal area]/area internal the IEL*100). Other plaque features were assigned a severity score (0 = absent, 1 = minimal, 2 = mild, 3 = moderate, 4 = marked, 5 = severe), including intimal necrosis/erosion, cartilage formation or calcification, intraplaque hemorrhage, thrombosis, intraplaque blood vessels, subacute intraplaque or perivascular inflammation, all assessed on H&E-stained sections. Using Movat’s pentachrome-stained slides, deposition of non-smooth muscle extracellular matrix (proteoglycan and/or collagen) within the tunica media and large areas of elastin loss were also scored in the same manner, 0–5. Elastin breaks (discontinuity in the elastic laminae) were counted per aortic root. Because of the greater availability of atheroma sections in the aortic root, any additional histological assessments described below were performed in these sections.

### Induction of advanced human-like plaque

Oscillatory shear stress-mediated atherosclerotic lesions were induced in the left carotid artery by partial carotid ligation (PCL). PCL is a surgical procedure that induces advanced human-like atherosclerotic lesions in atheroprone mouse models [21,22]. Briefly, under isoflurane (1–2%) anesthesia, the left carotid artery was exposed by blunt dissection. Three of the four branches of the left carotid artery, the external carotid artery distal to the superior thyroid artery, and the internal carotid and occipital arteries, were ligated using 6–0 suture. This allows for oscillatory shear and continued antegrade blood flow through the superior thyroid artery. An atherogenic, Paigen diet was initiated at the time of surgery and maintained for 5 weeks until atherosclerotic lesions were assessed in the left carotid artery. Euthanasia was performed in anesthetized mice (inhaled isoflurane) by exsanguination via cardiac puncture and verified by bilateral thoracotomy. The heart with attached aorta and carotid arteries were excised, formalin fixed and cleared of surrounding tissues with the aid of a dissecting microscope for analyses as described below. The area of spontaneous, lipogenic atherosclerotic plaques were also assessed in the descending aorta and aortic root of wildtype and *Arf6* heterozygous mice after consumption of Paigen diet for 5 weeks.

### Left carotid artery atheroma assessment

To assess the impact of modulating Arf6 expression on the size and properties of oscillatory shear stress-induced carotid artery plaques, samples were collected from WT and HET mice euthanized 5 weeks after partial carotid ligation (PCL) and initiation of the Paigen diet. Plaques in the left carotid artery were visualized and photographed with the aid of a dissecting microscope by Dr. Durrant. Two separate samples from the left carotid artery, proximal to the heart from the ligation site, containing atheromatous plaque, were excised, histologically prepared and H&E- and MT-stained for analyses based on human medical classification, plaque measurements, and histopathology scores as described for the aortic root. A section of the control, right carotid artery was also excised and prepared histologically. No plaques were found in the right carotid artery of any mouse.

### Plasma lipids

Total cholesterol, total triglycerides, LDL and HDL levels were measured using dedicated on-board reagents from Abbott Laboratories (Cat#7D62-21, 7D74-21, 1E31-20 and 3k33-22).

### Immune cell migration

To examine the effects of heterozygosity for *Arf6* on immune cell migration, total T cells were isolated from spleens of WT and HET mice using a T cell Isolation kit (EasySepTM Mouse T cell isolation kit, STEMCELL Technologies). To establish an effective dose of a chemokine C-C motif ligand 2 (CCL2) for subsequent migration assay in WT and HET cells, 100 μL of the immune cell suspension (2 X 106 total cells/ml in RPMI medium) from WT mice was added to the upper chamber of each 24 well transwell pre-coated with fibronectin (6.5mm diameter insert, 5μM pore size, Corning). 600 μL of RPMI medium with 0, 12.5, 25 and 50 ng/ml of CCL2 (Sigma) were added to the lower chamber. The transwell plates were incubated at 37°C, 5% CO_2_ for 3 hours. The migrated T cells in the lower chamber were collected and stained with anti-CD3+, anti-CD4+, anti-CD8+ and counted using FACs (Canto, BD science). We found that there was a 42–55% increase in cell migration in response to 25 ng/mL CCL2 with no further increase in migration at 50 ng/mL; thus, we utilized 25 ng/mL in the subsequent assays.

In addition, immunohistochemistry (IHC) was used to assess the presence of T cells and macrophages within and external to the plaque. Aortic root slides were sectioned at ~4 μm and stained for CD3 (T cells) and F4/80 (macrophages). Staining was conducted on the Leica Bond RX/RXm platform using standard chromogenic methods. For CD3, antigen retrieval was performed with pH-9 EDTA buffer, followed by primary antibody incubation (1:400, rat CD3-12 clone; Bio-Rad MCA14777) for 45 minutes at room temperature. For F4/80, antigen retrieval was performed with proteinase K buffer, followed by primary antibody incubation (1:400 rat CI: A3-1 clone; Bio-Rad MCA497R) for 30 minutes at room temperature. For each marker, an HRP-conjugated anti-rat secondary antibody was used for antibody binding detection with visualization via diaminobenzidine application and hematoxylin counterstain. The presence of these cells within histological sections of atheromas was assessed by a blinded pathologist and assigned a score according to a scale of 0–5 with 0 = absent or within normal limits/no labeling, 1 = minimal, 2 = mild, 3 = moderate, 4 = marked, 5 = severe.

### Cell proliferation

Electric Cell Impedance Sensing (ECIS) was used to assess proliferation in isolated endothelial and vascular smooth muscle cells from WT and HET mice as previously described [30,31]. Primary lung ECs were isolated using an optimized method. In brief, mouse lungs were minced using scissors, digested by collagenase-I and filtered through a 70μm strainer. Cell suspensions were incubated with PECAM conjugated dynabeads. Further, cells were seeded in 100mm culture dishes at 37  C in the incubator. When the cells were confluent, the cell suspension was sorted using ICAM conjugated dynabeads. The sorted cells were plated into 100mm dishes. Confluent cells were used functional experiments. Primary vascular smooth muscle cells were isolated from aorta as previously described [22]. Briefly, the thoracic aorta was cleaned of perivascular fatty tissue and digested in an enzyme solution (HBSS buffer with 1 mg/ml collagenase II, 1 mg/ml soybean trypsin Inhibitor, 0.744 unit/ml elastase, and 1% penicillin/streptomycin) for 10 minutes. After incubation, the adventitia was removed, the aorta opened longitudinally, and the endothelial cells detached by gently scrapping with a sterilized cotton swab. The aorta was minced and incubated with enzyme solution at 37°C for an additional 1 hour. After the incubation, the digestive enzyme solution was neutralized by isolation medium (DMEM/F12 with FBS and Penicillin/streptomycin), the cells were suspended in complete VSMC growth medium (DMEM/F12 with FBS, penicillin/streptomycin and L-glutamine) and plated. VSMCs were cultured in growth medium, and cells were passaged 3 times before the cell assays. Cell proliferation was measured in real time using Electric Cell-substrate Impedance Sensing (ECIS). Briefly, ECs were grown on 96-well array (96W20idf). Arrays were pre-coated with 10 μg/mL fibronectin for 30 minutes at 37°C, washed with PBS, and then 1X10^4^ ECs were seeded in EC growth medium (EBM-2 with FBS, hydrocortisone, hFGF, VEGF, R3-IGF, hEGF, GA, and Heparin). Data collected from each group was observed in triplicate. Data are presented as the average resistance over the first minute of the hour from 0 to 100 hours.

### Statistical analyses

To determine differences in mean values between genotypes, unpaired Student’s T-Tests were performed unless otherwise stated in the figure legends. For scaled data, Mann-Whitney tests were also used to determine differences for scaled variables such as atherosclerotic plaque grade and histological scoring of plaque characteristics. Figures are mean ± SEM with individual datapoints and p values. Results of the non-parametric statistical analyses are provided in the S1–S3 Tables. Significance was set at P≤0.05.

## Results

### Atherogenic stimulus increases Arf6 expression

**Fig 1:**
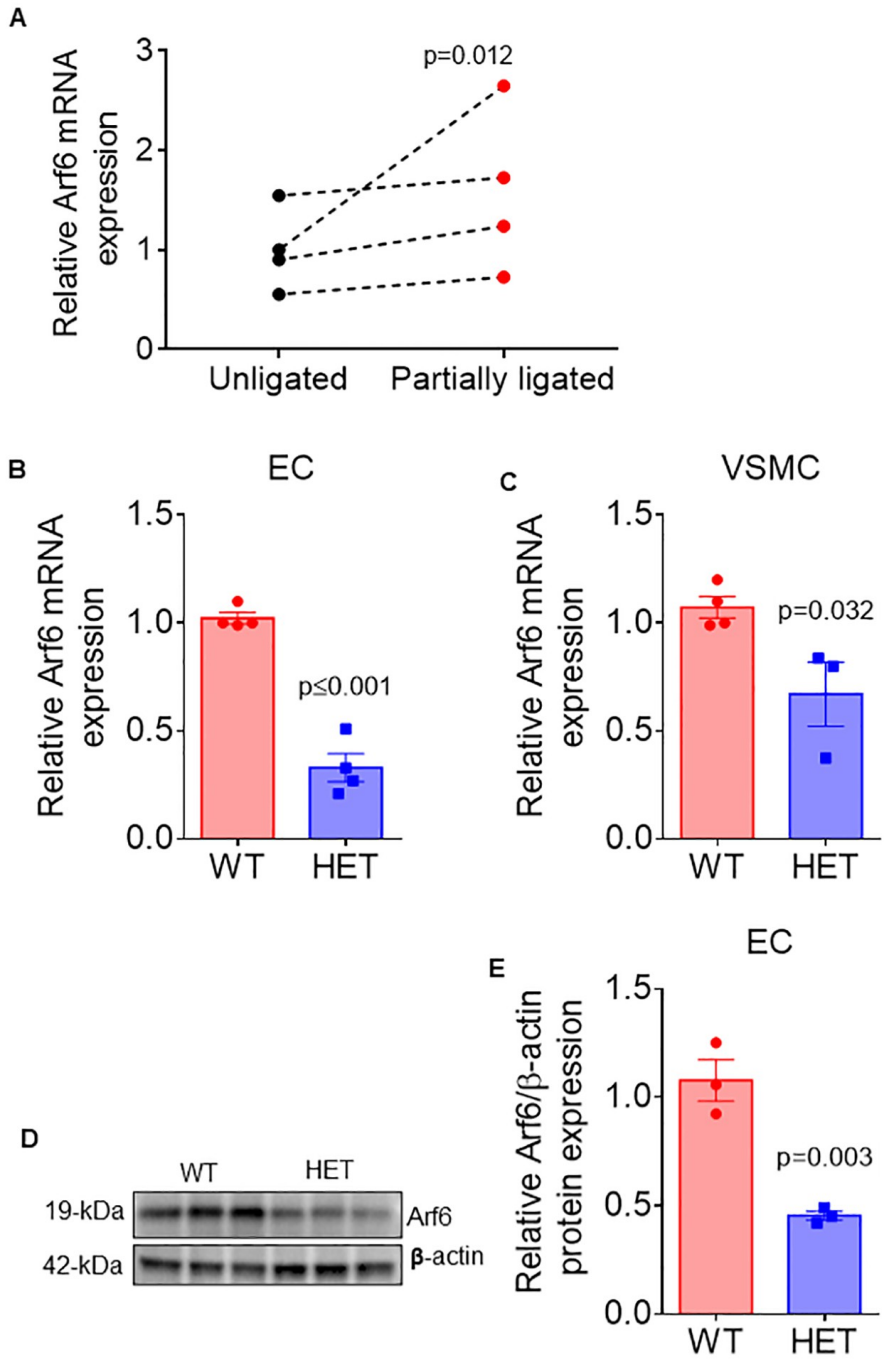
Low oscillatory shar stress *Arf6* heterozygosity decreases Arf6 expression. (A) Gene expression of *Arf6* in unligated (right) and partially ligated (left) carotid arteries, (B,C) Gene expression fo *Arf* from endothelial cell (EC) enriched and vascular smooth muscle cell (VSMC) enriched carotid artery lysates from wildtype (WT) and *Arf6* heterozygote (HET) mice (N = 3-4/group), (D) primary lung EC protein expression fo Arf6 and β-actin from WT and *Arf6* HET mice, (E) densitometric quantification of western blotting data. (N = 3/replicate, ECs for each replicate were pooled from lungs of 3–5 mice). Data are presented as individual pairs of unligated and partially ligated datapoints (A) or mean ± SEM with individual datapoints (B-E). Group differences were assessed using paired (A) or unpaired (B-E) student’s *t* test.

We first examined if atherogenic stimulus increases Arf6 expression in wildtype mice. To do so, we induced low oscillatory shear stress, a robust atherogenic stimulus, using partial carotid ligation surgery. We found that oscillatory shear stress increased Arf6 gene expression in the partially ligated left carotid artery as compared to unligated right carotid artery (Fig 1A; p = 0.012). This result suggests that elevated Arf6 expression may play a role in atherosclerosis.

### Heterozygosity reduces tissue expression of Arf6

We next examined if *Arf6* heterozygosity results in blunted gene and protein expression of Arf6 in tissues involved in the development of atherosclerosis. We found that *Arf6* mRNA expression was lower in endothelial cell- (Fig 1B; P<0.001) and smooth muscle-enriched (Fig 1C; P = 0.03) carotid artery lysates in HET compared to WT mice. We also found lower Arf6 protein in lysates from isolated primary lung endothelial cells (Fig 1D and 1E; P = 0.003). Taken together, these data demonstrate proof of concept that germline *Arf6* heterozygosity reduces gene and protein expression in this experimental mouse model.

### Arf6 heterozygosity reduces the area of spontaneous atheromas in the descending aorta and aortic root as well as the severity of atheromas in the aortic root

**Fig 2:**
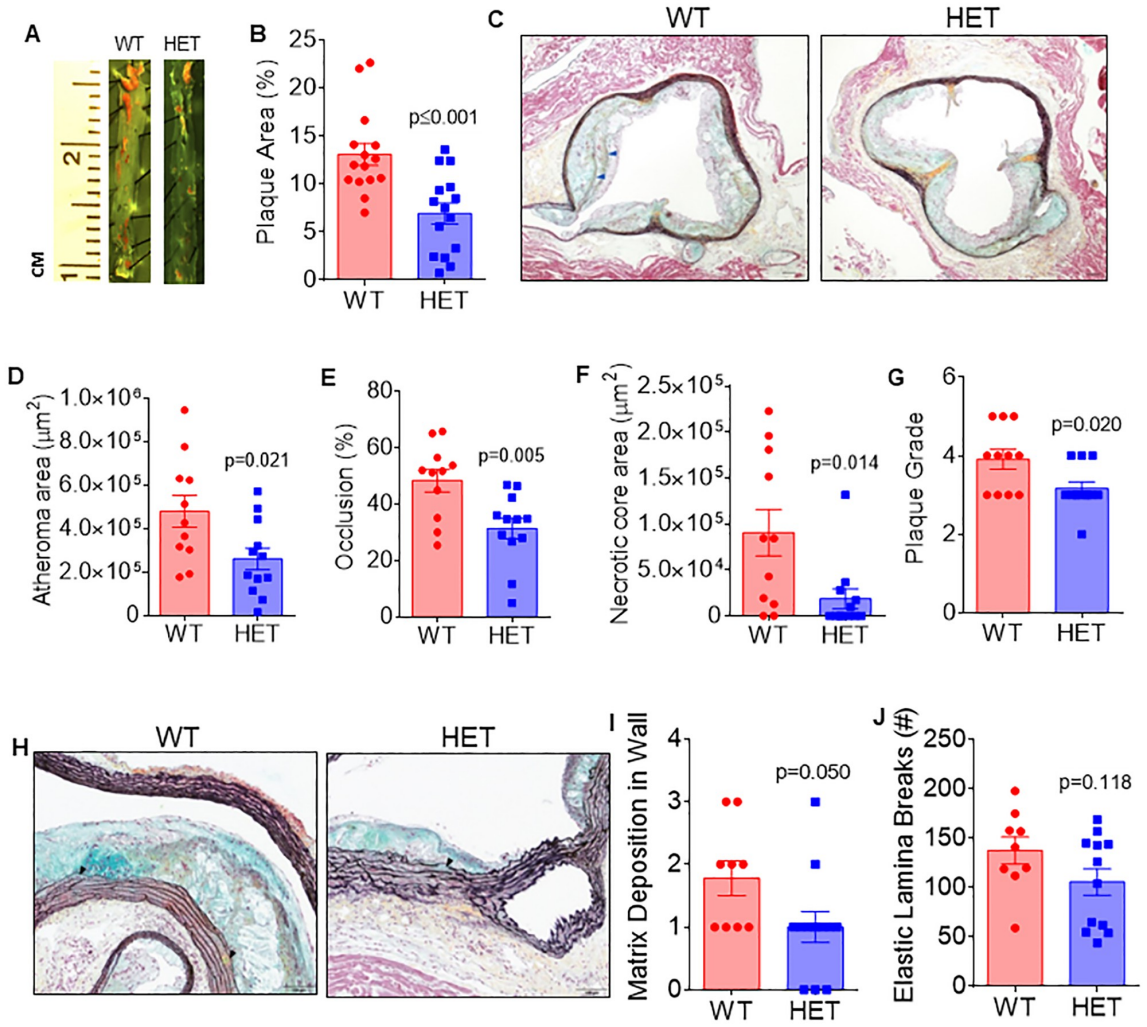
*Arf6* heterozygosity attenuates burden and severity of spontaneous atherosclerotic plaque. (A, B) representative images and quantification of descending aortic plaque from wildtype (WT) and *Arf6* heterozygote (HET) mice (N = 15/group), (C) representative images of Movat’s pentachrome stained aortic root sections for atheroma and necrotic core areas, luminal occlusion and plaque grades. Note: The fibrous caps overlying necrotic cores in the WT sample (blue arrowheads), (D) aortic root atheroma area (N = 11/group), (E) percent luminal occlusion of aortic root (n = 11-12/group), (F) area of necrotic core (1 = 11/group) and (G) plaque grade (N = 11/group). (H) Representative images of Movat’s pentachrome stained aortic root sections for non-muscular matrix (teal or yellow color) and the elastic laminae (black lines). (I) Matrix deposition score (N = = 9/group) and (J) counts of elastin lamina breaks in WT and Arf6 HET mice(N = 9/group). Data are presented as mean ± SEM with individual datapoints. Scale bars indicate 100 μm. Group differences were assessed using unpaired student’s *t* test for continuous variables and Mann-Whitney tests for scaled variables.

We assessed if lower Arf6 expression attenuates atherosclerotic plaque burden in the thoracic aorta after atherosclerotic diet consumption. Mice were fed atherogenic diet for 5–8 week. There was no effect of the number of weeks on diet (P = 0.474) or significant interaction between weeks on diet and genotype (P = 0.225), but there was a main effect for genotype (P˂0.001) such that HET mice demonstrated a lower aortic plaque burden compared to WT mice (Fig 2A and 2B).

Next, we examined the size and severity of aortic root atheromas using Movat’s pentachrome stained aortic root sections (Fig 2C and 2H). We found that aortic root atheromas were present in all mice; however, atheroma area was 46% smaller in HET compared to WT mice (Fig 2D; P = 0.021). Percent luminal occlusion was higher in WT compared to HET mice (Fig 2E; P = 0.005). Necrotic cores were present in 81% (9 of 11) atheromas from WT mice, but only 42% (5 of 12) of HET mice. The size of the necrotic core ranged from 0% (i.e., not present) to 35% of the atheroma in WT mice and from 0% to 30% of the atheroma in HET mice. The size of the necrotic core was smaller in HET compared to WT mice when compared as absolute area (Fig 2F; P = 0.014).

Further analysis indicated that plaque severity, assessed by plaque grade, was lower in HET compared to WT mice (Fig 2G; P = 0.020). Moreover, we found a reduction in matrix deposition (Fig 2I; P = 0.05) and a tendency for fewer breaks in the elastic lamina of HET compared to WT mice (Fig 2J; P = 0.118), indicating better maintenance of vascular wall morphology in the HET mice. S1 Table provides descriptive statistics for scaled variables, as well as p values from non-parametric, Mann-Whitney tests of median differences for these data. Taken together, these data suggest that Arf6 heterozygosity attenuates atherosclerotic burden and may reduce severity.

### Arf6 heterozygosity reduces atheroma size and severity in advanced human-like carotid artery plaques induced by acutely increased oscillatory shear stress

**Fig 3:**
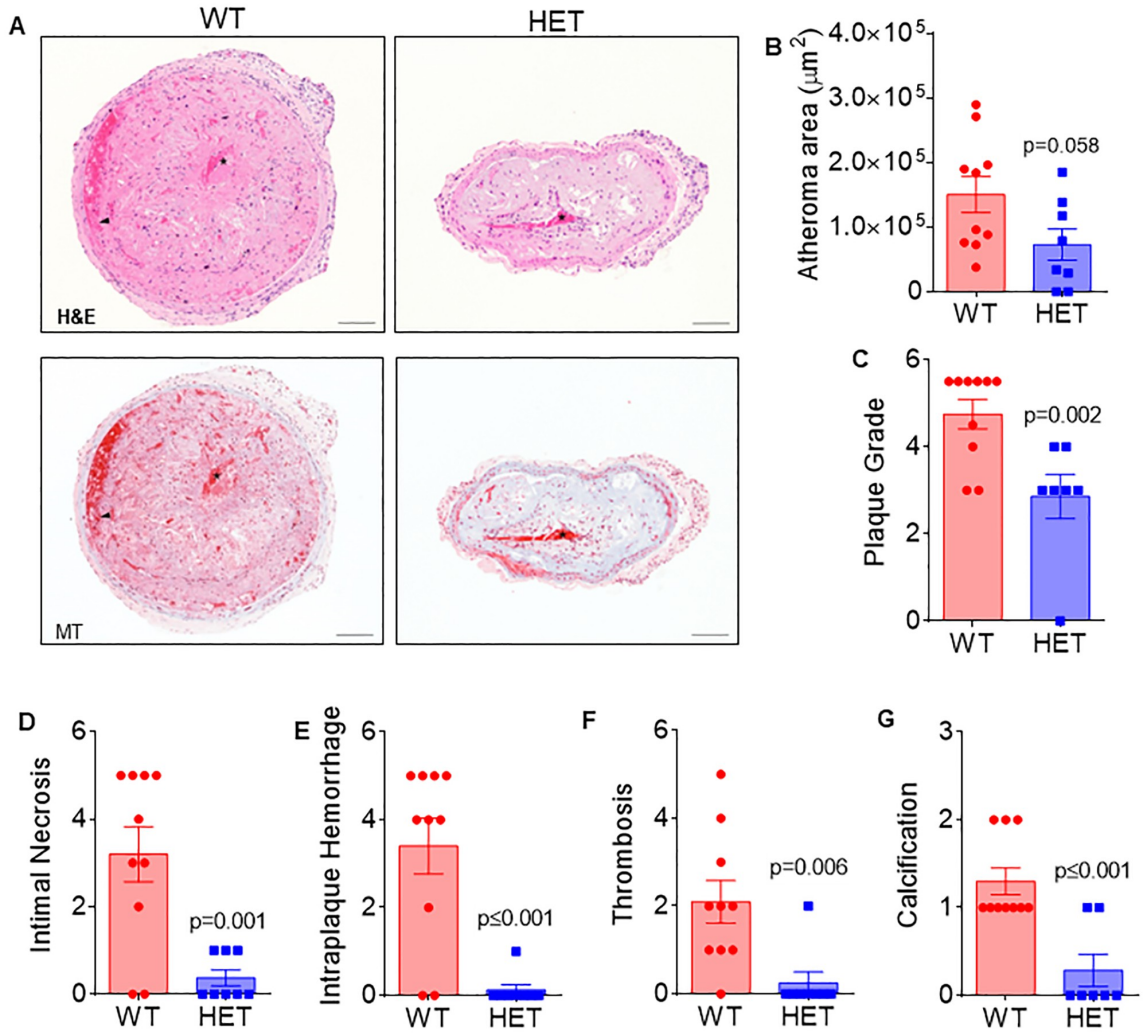
*Arf6* heterozygosity attenuates the size and severity of oscillatory shear stress induced advanced human-like carotid artery atherosclerotic plaque. Quantification and scoring of left carotid artery sections after five weeks of partial carotid ligation surgery and atherogenic diet consumption (A) Representative images of hematoxylin and eosin as well as Masson’s trichrome stained left carotid artery sections. The arterial lumen is indicated by *. In the WT sample, there is loss of the endothelial lining, and a luminal thrombus is present. Hemorrhage within the plaque is also present (black arrowhead), (B) total atheroma area (N = 8-10/group), (C) plaque grade (N = 7-10/group) (D) intimal necrosis (N = 8-10/group), (E) intraplaque hemorrhage (N = 8-10/group), (F) thrombosis (N = 8-10/group), and (G) calcification (N = 8-10/group), of WT and Arf6 HET mice. Data are presented as mean ± SEM with individual datapoints. Scale bars indicate 100 μm. Group differences were assessed using unpaired student’s *t* test for continuous variables and Mann-Whitney tests for scaled variables.

We next sought to examine the impact of Arf6 heterozygosity on advanced plaque characteristics such as intimal necrosis, intraplaque hemorrhage, thrombosis, and calcification. Although spontaneously developed murine plaques lack these features of advanced complex human atherosclerotic plaques, this limitation can be reduced by performing partial carotid ligation (PCL) which induces acute oscillatory shear stress in the carotid artery. Five weeks of atherogenic diet consumption after PCL surgery led to the development of human-like continuous advanced plaques in the left carotid artery, allowing us to examine the advanced plaque characteristics using H&E and MT-stained sections (Fig 3A). Atheromas were present in 100% of WT (6 of 6 mice, 10 of 10 sections) and ~80% of HET (4 of 5 mice, 5 of 7 sections) mice. Although it failed to reach significance (P = 0.058), atheroma area was 48% smaller in the left carotid arteries of HET compared to WT mice (Fig 3B), which is in agreement with atheromas observed in the aortic root.

We found that plaque grade (Fig 3C; P = 0.002) and scoring for intimal necrosis (Fig 3D; P = 0.001), intraplaque hemorrhage (Fig 3E; P<0.001), thrombosis (Fig 3F; P = 0.006) and calcification (Fig 3G; P = 0.090) were all lower in PCL-induced carotid artery lesions from HET compared to WT mice. S2 Table provides descriptive statistics for all scaled variables, as well as p values from non-parametric, Mann-Whitney tests of median differences for these data. Together with the results in the aortic root, these data indicate that reducing *Arf6* lowers susceptibility to and severity of atherosclerotic lesions.

### Atheroprotection afforded by Arf6 heterozygosity is independent of changes in circulating lipids

**Fig 4:**
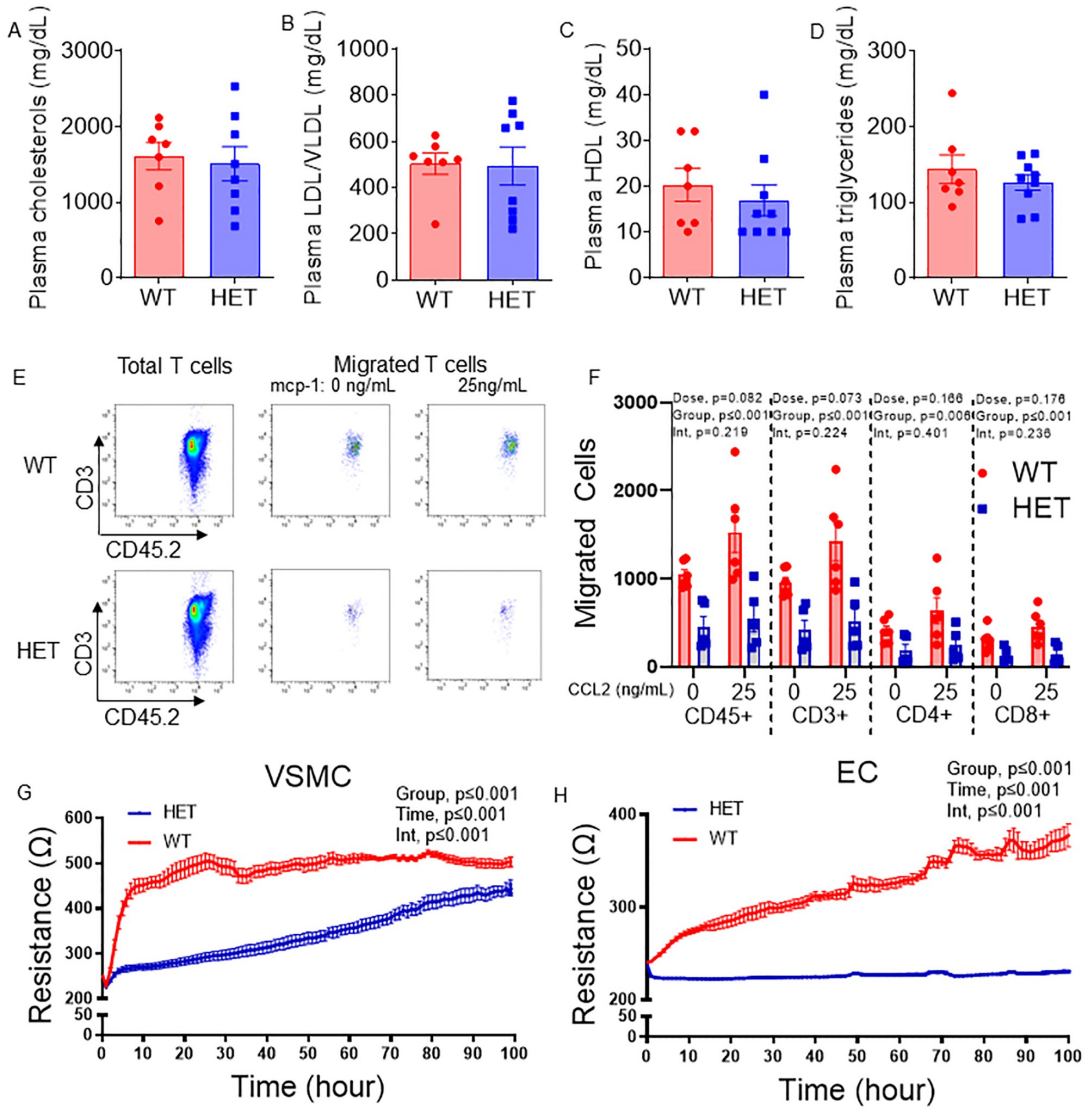
*Arf6* heterozygosity does not alter plasma lipids but reduces immune cell migration as well as endothelial and smooth muscle cell proliferation. (A) Plasma total cholesterols (N = 7-8/group), (B) plasma low-density and very low-density lipoprotein (LDL/VLDL) (N = 7-8/group), (C) plasma high-density lipoprotein (HDL) (N = 7-8/group), (D) plasma triglycerides (N = 7-8/group). (E) Representative images of characterization and counting of total and migrated T cells using flow cytometry, (F) baseline and C-C motif ligand 2 (CCL2)-induced migration of lymphocytes, total T cells, helper T cells and cytotoxic T cells (N = 6/replicate) (G, H) *in vitro* resistance, a measure of proliferation, of primary vascular smooth muscle cell (VSMC) and lung endothelial cell (EC). N = 6/replicate for SMC proliferation and 3/replicate for EC proliferation. SMC and EC for each replicate were pooled from thoracic aorta and lungs of 3–5 mice, respectively. Data are presented as mean ± SEM. Group differences were assessed using unpaired student’s *t* test (lipid profile), two-way ANOVA (T cell migration) or repeated measure ANOVA (EC and VSMC proliferation).

To determine if alterations in circulating lipids contribute to the atheroprotection afforded by Arf6 heterozygosity, we measured total, LDL/VLDL and HDL cholesterol as well as circulating triglycerides in WT and HET mice after 5 weeks on atherogenic diet. Heterozygosity for Arf6 did not alter circulating cholesterol (Fig 4A–4D; all P≥0.40), suggesting that the reduction in plaque burden and improvement in plaque characteristics in Arf6 HET mice are independent of circulating lipid profile.

### Reduced immune cell migration as well as reduced vascular smooth muscle cell and endothelial cell proliferation may contribute to atheroprotection

To explore a potential role of altered immune cell migration and vascular smooth muscle cell and endothelial cell proliferation in atheroprotection afforded by *Arf6* heterozygosity, we performed in vitro migration and proliferation assays. There was a significant main effect for group with HET mice being lower than WT mice for migration of CD45+ (P<0.001), CD3+ (P<0.001), CD4+ (P = 0.006) and CD8+ (P<0.001) cells (Fig 4E and 4F), suggesting Arf6 decreases immune cell migration. To examine immune cell migration into the atherosclerotic plaque, we performed immunohistochemical staining for macrophages and T cells. We found that intraplaque macrophages were significantly lower in Arf6 HET as compared to WT mice (S3 Table; p = 0.05). No difference was found in external macrophages between groups (S3 Table; p = 0.57). Intraplaque T cells were not different between groups (p = 0.70); however arterial T cells external to plaque were marginally lower in HET compared to WT mice (S3 Table; p = 0.07). We also found that proliferation, as measured by resistance of cell monolayer over time, of primary aortic smooth muscle and lung endothelial cells were lower (Fig 4G and 4H; both p≤0.001) from *Arf6* HET compared to WT mice. These data suggest that attenuated immune cell migration and endothelial and vascular smooth muscle cell proliferation may contribute to suppressing atherosclerosis in Arf6 HET mice.

## Discussion

The aim of this study was to elucidate the role of Arf6 in atherosclerotic plaque burden and severity. The key novel findings of this present study are as follows: 1) Arf6 heterozygosity reduces the burden and severity of spontaneous atherosclerotic plaque in the descending aorta and in the aortic root; 2) Arf6 heterozygosity attenuates the size and severity of oscillatory shear stress induced advanced human-like carotid artery atherosclerotic plaque; 3) *in vitro* T cell migration as well as VSMC and EC proliferation are attenuated in cells from Arf6 heterozygous mice; and 4) the lower plaque burden and improved plaque quality in Arf6 heterozygous mice is independent of changes in the circulating lipid profile. Taken together, these results suggest that the reductions in atherosclerotic plaque burden and improvements in plaque quality due to lower Arf6 expression may result from alterations in the ability to sense/respond to pro-atherogenic stimuli as well as reduced cell proliferation and migration. However, alterations in circulating lipids do not appear to play a significant role. To our knowledge, this is the first study to demonstrate the role of Arf6 in atherosclerotic disease progression and our findings support exploration of pharmacological Arf6 inhibition as a therapeutic strategy in atherosclerotic disease.

Atherosclerotic plaques form in the large arteries in the face of dyslipidemia and at regions of low and oscillatory shear stress, such as branch points and curvatures [2,32]. In this current study, we demonstrate that reduction of Arf6 attenuates atherosclerotic plaque burden in mice. Arf6 is a GTPase that is active when it is bound to GTP and inactive when it is bound to GDP [14]. When active, Arf6 regulates a host of cellular and molecular process including, but not limited to, cell proliferation, migration, and actin/cytoskeleton reorganization [14]. In the endothelial cell, Arf6 senses the flow patterns and shear stress via vascular endothelial protein tyrosine phosphatase, which subsequently lead to actin polymerization and morphological changes [18]. Thereby, the atheroprotection in Arf6 heterozygous mice may partly stem from the attenuated ability of endothelial cells to sense oscillatory shear stress. Although we cannot differentiate between spontaneous plaque and oscillatory shear stress-induced plaque in the aorta, the attenuated size and severity of left carotid artery atheromas provide direct evidence that Arf6 reduction remarkably suppresses oscillatory shear stress-induced atherosclerosis.

Beyond the size of atherosclerotic lesions, we also found improvements in plaque severity to be afforded by Arf6 heterozygosity. Presence of a necrotic core was less frequent and, when present, tended to be smaller in HET compared to WT mice. Arf6 heterozygosity resulted in a less severe plaque grade at both the aortic root and carotid artery. Luminal occlusion, intimal necrosis, intraplaque hemorrhage, thrombosis, and calcification were attenuated by Arf6 heterozygosity in the advanced human-like carotid artery atheromas, suggesting a less severe plaque. In the course of human atherosclerotic plaque development, formation of necrotic core and calcification occurs in the advanced plaque leading to the occlusion of the arterial lumen [2]. As the plaque grows, it forms neo vessels known as vasa vasorum that feed the plaque and promote immune cells infiltration [2,33]. Vasa Vasorum, a rupture-prone single layer of endothelial cells lacking surrounding tissues, also promotes hemorrhage and thrombosis contributing to the vulnerability of plaque [34]. In the face of hemodynamic forces, when a vulnerable plaque gets dislodged, it results in clinical cardiovascular events such as myocardial ischemia and infarction, stroke, and paralysis [2,34]. Our study supports that Arf6 inhibition can be an effective strategy to modulate these advanced plaque characteristics.

Cell migration and proliferation plays critical role throughout the entire progression of atherosclerosis [35–37]. Here, we found that Arf6 heterozygosity reduces the migration of T cells both basally and in response to stimulation by macrophage chemoattractant protein-1. We also demonstrate that Arf6 heterozygosity attenuates the proliferation of vascular smooth muscle and endothelial cells. These findings suggest that immune cell migration as well as endothelial and vascular smooth muscle cell proliferation are important contributors to atheroprotection in Arf6 heterozygous mice. The role of Arf6 in cell migration and proliferation has been previously elucidated in several cell types and our findings are in agreement with those studies. For example, Arf6 is a critical regulator of migration of epithelial cells [38], endothelial cells [39], HEK293 cell and vascular smooth muscle cells [40,41]. Also, evidence exists that Arf6 promotes VEGF-mediated EC proliferation and angiogenesis [15,39,42]. Mechanistically, Arf6 regulates cell migration and proliferation via Rac1 GTPase protein. Arf6 and Rac1 form a transient complex leading to the production of reactive oxygen species and phosphorylation of VEGF receptor 2 (VEGFR2) and caveolin-1, resulting in actin and cytoskeleton reorganization, receptor trafficking, membrane shuffling, and thus migration and proliferation [39]. Thus, our findings are supported by the existing literature as well as display novel evidence that suppressed cell migration and proliferation contributes to atheroprotection in Arf6 HET mice.

Elevated triglycerides and cholesterols induce endothelial dysfunction leading to LDL infiltration and oxidation in the subintimal layer, triggering monocyte infiltration and differentiation into macrophages [1,2]. Differentiated macrophages engulf oxidized LDL and form foam cells resulting in vascular smooth muscle cell migration and proliferation, platelet aggregation, and sustained inflammation, all of which contribute to the progression of atherosclerosis [1,2]. Our study demonstrates that the atheroprotection in Arf6 heterozygous mice was independent of changes in systemic dyslipidemia and associated with shear sensing, cell migration and proliferation. Indeed, evidence exist that targeting these cell behaviors can suppress atherosclerosis independent of systemic lipids. For example, a recent study demonstrated that inhibition of endothelial mechanosensitive protein, TXNDC5, reduces atherosclerosis without affecting plasma cholesterols [43]. Likewise, treatment with tetrahydrobiopterin improved endothelial cell function and improved atherosclerosis in *ApoE*^*-/-*^ mice independent of plasma cholesterols [44]. Sano et. al., [45] demonstrated that platelet-derived growth factor β (PDGF-β) promotes smooth muscle proliferation resulting in atheroprogression and inhibition of PDGF-β suppressed atherosclerosis in *ApoE*^*-/-*^ mice without altering plasma lipids. Taken together, the notion of targeting cholesterol-independent mechanisms in atherosclerosis is supported by existing literature and our study identifies a novel mediator that can suppress atherosclerosis without affecting plasma cholesterols.

## Conclusion and future directions

In summary, our findings demonstrate that Arf6 heterozygosity suppresses atherosclerotic plaque development and severity. Modulation to the endothelial cell’s ability to sense oscillatory shear stress, cell migration and proliferation may be the primary mediators in the atheroprotection afforded by Arf6 heterozygosity. Future studies should also explore the role of Arf6 in specific tissues and cell types, to both provide mechanistic insight and direct the development of targeted therapeutics. This study supports the exploration of pharmacological Arf6 inhibition to attenuate the burden and severity of atherosclerosis. Previous studies demonstrate that inhibition of Arf6 is an effective strategy to treat a host of vascular pathologies [14,15,42,46]. Pharmacological Arf6 inhibitors, such as Navigen-2729, are commercially available and have demonstrated efficacy in a variety of preclinical disease models [14]. Studies are also warranted to examine the effects of Arf6 inhibition in other cardiovascular disease-related comorbidities such as metabolic dysfunction. Genetically modified mouse models and tissue or organ targeted delivery of pharmacological Arf6 inhibitors can be instrumental for such future investigations.

## Supporting information

S1 Fig Original uncropped blots. Primary lung EC protein expression of Arf6 and β-actin from WT and *Arf6* HET mice. The cropped versions of these blots are shown in Fig 1D.
(PPTX)

S1 Table Plaque grade and scoring for aortic root matrix deposition. Aortic roots were collected after 5–8 weeks on atherogenic diet, fixed, and paraffin embedded for histological sectioning and analysis. Arterial sections containing an atheroma were stained with H&E or Movat’s pentachrome and plaque grade and characteristics were evaluated by a blinded, ACVP-board-certified veterinary pathologist. Severity score of the plaques was determined by assigning a plaque grade based on AHA classifications. This grade is based on a scale of 1–7 with 1 = intimal thickening, 2 = intimal xanthoma, 3 = pathological intimal thickening, 3.5 = intimal thickening with erosion, 4 = fibrous cap atheroma, 4.5 = fibrous cap atheroma with erosion, 5 = thin fibrous cap atheroma, 5.5 plaque rupture, 6 = calcified nodule, 7 = fibrocalcific plaque. All other measures are on a scale of 0–5 with 0 = absent or within normal limits/no labeling, 1 = minimal, 2 = mild, 3 = moderate, 4 = marked, 5 = severe. Min: Minimum score. Max: Maximum score. P value from Mann-Whitney nonparametric test.
(DOCX)

S2 Table Plaque grade and scoring for atheroma characteristics in the left carotid artery after partial carotid ligation (PCL). Carotid arteries were collected five weeks after PCL and initiation of an atherogenic diet. Arteries were fixed and paraffin embedded for histological sectioning and analysis. Arterial sections containing an atheroma were stained with H&E or Masson’s trichrome and plaque grade and characteristics were evaluated by a blinded, ACVP-board-certified veterinary pathologist. Severity score of the plaques was determined by assigning a plaque grade based on AHA classifications. This grade is based on a scale of 1–7 with 1 = intimal thickening, 2 = intimal xanthoma, 3 = pathological intimal thickening, 3.5 = intimal thickening with erosion, 4 = fibrous cap atheroma, 4.5 = fibrous cap atheroma with erosion, 5 = thin fibrous cap atheroma, 5.5 plaque rupture, 6 = calcified nodule, 7 = fibrocalcific plaque. N is the number of animals per group and n is the total number of sections evaluated. Other plaque findings were assigned a severity score (0 = absent, 1 = minimal, 2 = mild, 3 = moderate, 4 = marked, 5 = severe). Min is minimum score. Max is maximum score. P value from Mann-Whitney nonparametric test.
(DOCX)

S3 Table Assessment of immune cells within and adjacent to aortic root atheromas from wildtype (WT) and *Arf6* heterozygous (HET) mice. Aortic roots were collected, fixed and paraffin embedded for histological sectioning and analysis. Immunohistochemical F4/80 and CD3 staining was used to evaluate the abundance of macrophages and T cells both internal (intraplaque) and external to the atheroma. Immunolabeling was scored on a scale of 0–5 with 0 = absent or within normal limits/no labeling, 1 = minimal/focal labeling, 2 = mild/small aggregates, 3 = moderate/multifocal aggregates, 4 = marked/large or regionally extensive aggregates, 5 = severe//diffuse immunolabeling of compartment. N is the number of animals per group and n is the total number of sections evaluated. Min: Minimum score. Max: Maximum score. P value from Mann-Whitney nonparametric test.
(DOCX)
