# Supplementary figures and images for: Heterozygosity for ADP-ribosylation factor 6 suppresses the burden and severity of atherosclerosis
Source: PLoS One. 2023 May 10;18(5):e0285253. doi: 10.1371/journal.pone.0285253 (PMC10171652; doi:10.1371/journal.pone.0285253)

## Slide 1
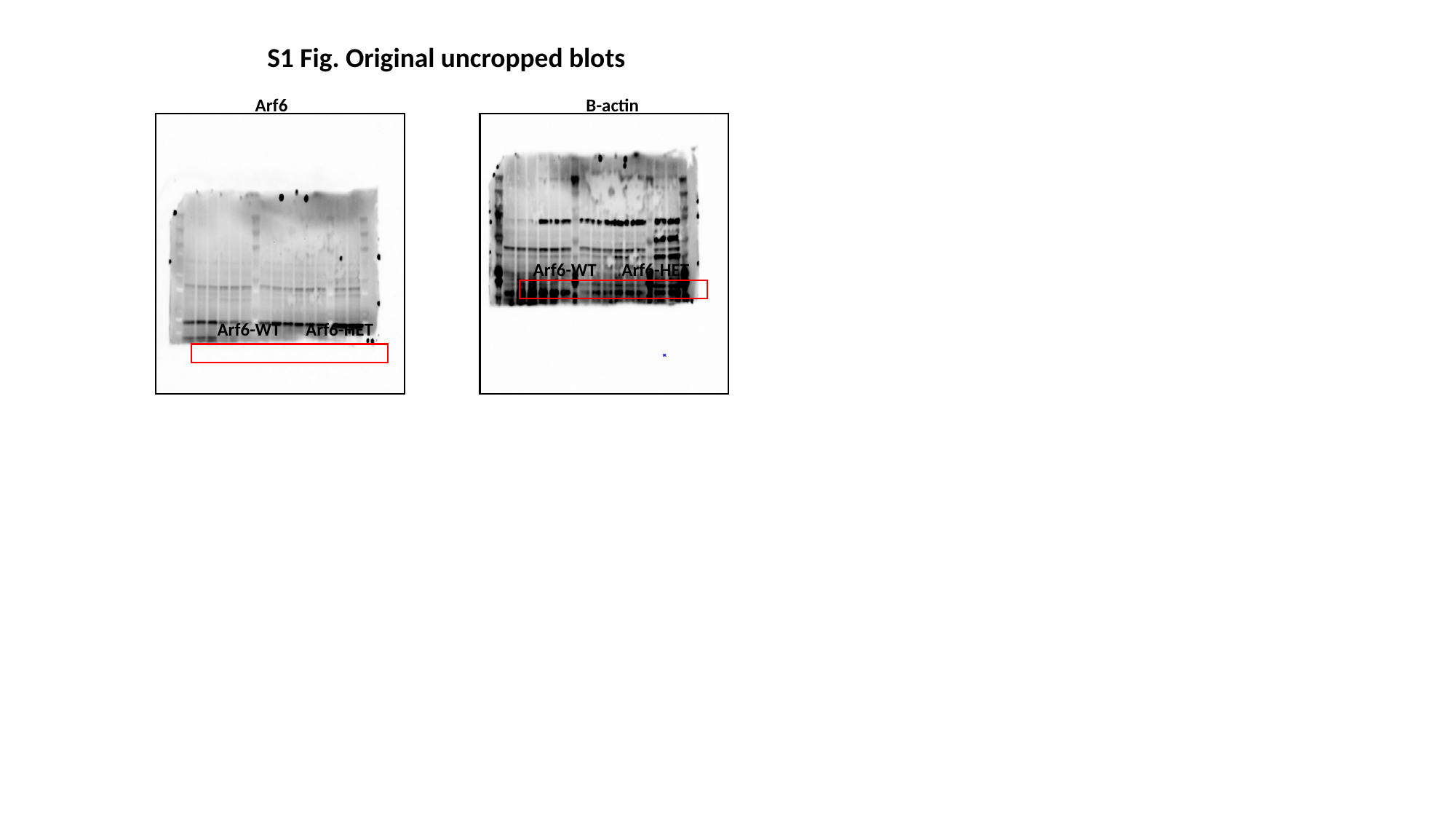

S1 Fig. Original uncropped blots
Β-actin
Arf6
Arf6-WT Arf6-HET
Arf6-WT Arf6-HET

Supplement: S1 Fig — Primary lung EC protein expression of Arf6 and β-actin from WT and Arf6 HET mice. The cropped versions of these blots are shown in Fig 1D. (PPTX) [file pone.0285253.s001.pptx]
