# Supplementary material for: Heterozygosity for ADP-ribosylation factor 6 suppresses the burden and severity of atherosclerosis
Source: PLoS One. 2023 May 10;18(5):e0285253. doi: 10.1371/journal.pone.0285253 (PMC10171652; doi:10.1371/journal.pone.0285253)
Supplement: S1 Table — Aortic roots were collected after 5–8 weeks on atherogenic diet, fixed, and paraffin embedded for histological sectioning and analysis. Arterial sections containing an atheroma were stained with H&E or Movat’s pentachrome and plaque grade and characteristics were evaluated by a blinded, ACVP-board-certified veterinary pathologist. Severity score of the plaques was determined by assigning a plaque grade based on AHA classifications. This grade is based on a scale of 1–7 with 1 = intimal thickening, 2 = intimal xanthoma, 3 = pathological intimal thickening, 3.5 = intimal thickening with erosion, 4 = fibrous cap atheroma, 4.5 = fibrous cap atheroma with erosion, 5 = thin fibrous cap atheroma, 5.5 plaque rupture, 6 = calcified nodule, 7 = fibrocalcific plaque. All other measures are on a scale of 0–5 with 0 = absent or within normal limits/no labeling, 1 = minimal, 2 = mild, 3 = moderate, 4 = marked, 5 = severe. Min: Minimum score. Max: Maximum score. P value from Mann-Whitney nonparametric test. (DOCX) [file pone.0285253.s002.docx]

| **Aortic Root** | **WT (N=9-11)** | | | |  | **HET (N=12)** | | | |  |
| --- | --- | --- | --- | --- | --- | --- | --- | --- | --- | --- |
|  | **Median** | **Min** | **Max** | **Mode** |  | **Median** | **Min** | **Max** | **Mode** | **P**  **value** |
| **Plaque Grade** | 4 | 3 | 5 | 3 |  | 3 | 2 | 4 | 3 | ***0.03*** |
| **Matrix Deposition** | 2 | 1 | 3 | 1 |  | 1 | 0 | 3 | 1 | ***0.04*** |

**S1 Table.** **Plaque grade and scoring for aortic root matrix deposition.** Aortic roots were collected after 5-8 weeks on atherogenic diet, fixed, and paraffin embedded for histological sectioning and analysis. Arterial sections containing an atheroma were stained with H&E or Movat’s pentachrome and plaque grade and characteristics were evaluated by a blinded, ACVP-board-certified veterinary pathologist. Severity score of the plaques was determined by assigning a plaque grade based on AHA classifications. This grade is based on a scale of 1-7 with 1=intimal thickening, 2=intimal xanthoma, 3=pathological intimal thickening, 3.5= intimal thickening with erosion, 4= fibrous cap atheroma, 4.5= fibrous cap atheroma with erosion, 5= thin fibrous cap atheroma, 5.5 plaque rupture, 6=calcified nodule, 7= fibrocalcific plaque. All other measures are on a scale of 0-5 with 0=absent or within normal limits/no labeling, 1=minimal, 2=mild, 3=moderate, 4=marked, 5=severe. Min: minimum score. Max: maximum score. P value from Mann-Whitney nonparametric test.
