# Supplementary material for: Heterozygosity for ADP-ribosylation factor 6 suppresses the burden and severity of atherosclerosis
Source: PLoS One. 2023 May 10;18(5):e0285253. doi: 10.1371/journal.pone.0285253 (PMC10171652; doi:10.1371/journal.pone.0285253)
Supplement: S2 Table — Carotid arteries were collected five weeks after PCL and initiation of an atherogenic diet. Arteries were fixed and paraffin embedded for histological sectioning and analysis. Arterial sections containing an atheroma were stained with H&E or Masson’s trichrome and plaque grade and characteristics were evaluated by a blinded, ACVP-board-certified veterinary pathologist. Severity score of the plaques was determined by assigning a plaque grade based on AHA classifications. This grade is based on a scale of 1–7 with 1 = intimal thickening, 2 = intimal xanthoma, 3 = pathological intimal thickening, 3.5 = intimal thickening with erosion, 4 = fibrous cap atheroma, 4.5 = fibrous cap atheroma with erosion, 5 = thin fibrous cap atheroma, 5.5 plaque rupture, 6 = calcified nodule, 7 = fibrocalcific plaque. N is the number of animals per group and n is the total number of sections evaluated. Other plaque findings were assigned a severity score (0 = absent, 1 = minimal, 2 = mild, 3 = moderate, 4 = marked, 5 = severe). Min is minimum score. Max is maximum score. P value from Mann-Whitney nonparametric test. (DOCX) [file pone.0285253.s003.docx]

**S2 Table.** **Plaque grade and scoring for atheroma characteristics in the left carotid artery after partial carotid ligation (PCL).** Carotid arteries were collected five weeks after PCL and initiation of an atherogenic diet. Arteries were fixed and paraffin embedded for histological sectioning and analysis. Arterial sections containing an atheroma were stained with H&E or Masson’s trichrome and plaque grade and characteristics were evaluated by a blinded, ACVP-board-certified veterinary pathologist. Severity score of the plaques was determined by assigning a plaque grade based on AHA classifications. This grade is based on a scale of 1-7 with 1=intimal thickening, 2=intimal xanthoma, 3=pathological intimal thickening, 3.5= intimal thickening with erosion, 4= fibrous cap atheroma, 4.5= fibrous cap atheroma with erosion, 5= thin fibrous cap atheroma, 5.5 plaque rupture, 6=calcified nodule, 7= fibrocalcific plaque. N is the number of animals per group and n is the total number of sections evaluated. Other plaque findings were assigned a severity score (0=absent, 1=minimal, 2=mild, 3=moderate, 4=marked, 5=severe). Min is minimum score. Max is maximum score. P value from Mann-Whitney nonparametric test.

| **Carotid Artery** | **WT (N/n=6/10)** | | | |  | **HET (N/n=5/8)** | | | |  |
| --- | --- | --- | --- | --- | --- | --- | --- | --- | --- | --- |
|  | **Median** | **Min** | **Max** | **Mode** |  | **Median** | **Min** | **Max** | **Mode** | ***p***  ***value*** |
| **Plaque Grade** | 5.5 | 3 | 5.5 | 5.5 |  | 3 | 0 | 4 | 0 | ***0.003*** |
| **Intimal Necrosis** | 3.5 | 0 | 5 | 5 |  | 0 | 0 | 1 | 0 | ***0.009*** |
| **Calcification** | 1 | 1 | 2 | 1 |  | 0 | 0 | 3 | 0 | ***0.03*** |
| **Intraplaque**  **Hemorrhage** | 4 | 0 | 5 | 5 |  | 0 | 0 | 1 | 0 | ***0.002*** |
| **Thrombosis** | 2 | 0 | 5 | 2 |  | 0 | 0 | 2 | 0 | ***0.004*** |
