# Supplementary material for: Heterozygosity for ADP-ribosylation factor 6 suppresses the burden and severity of atherosclerosis
Source: PLoS One. 2023 May 10;18(5):e0285253. doi: 10.1371/journal.pone.0285253 (PMC10171652; doi:10.1371/journal.pone.0285253)
Supplement: S3 Table — Aortic roots were collected, fixed and paraffin embedded for histological sectioning and analysis. Immunohistochemical F4/80 and CD3 staining was used to evaluate the abundance of macrophages and T cells both internal (intraplaque) and external to the atheroma. Immunolabeling was scored on a scale of 0–5 with 0 = absent or within normal limits/no labeling, 1 = minimal/focal labeling, 2 = mild/small aggregates, 3 = moderate/multifocal aggregates, 4 = marked/large or regionally extensive aggregates, 5 = severe//diffuse immunolabeling of compartment. N is the number of animals per group and n is the total number of sections evaluated. Min: Minimum score. Max: Maximum score. P value from Mann-Whitney nonparametric test. (DOCX) [file pone.0285253.s004.docx]

**S3 Table.** **Assessment of immune cells within and adjacent to aortic root atheromas from wildtype (WT) and *Arf6* heterozygous (HET) mice.** Aortic roots were collected, fixed and paraffin embedded for histological sectioning and analysis. Immunohistochemical F4/80 and CD3 staining was used to evaluate the abundance of macrophages and T cells both internal (intraplaque) and external to the atheroma. Immunolabeling was scored on a scale of 0-5 with 0=absent or within normal limits/no labeling, 1=minimal/focal labeling, 2=mild/small aggregates, 3=moderate/multifocal aggregates, 4=marked/large or regionally extensive aggregates, 5=severe//diffuse immunolabeling of compartment. N is the number of animals per group and n is the total number of sections evaluated. Min: minimum score. Max: maximum score. P value from Mann-Whitney nonparametric test.

| **Aortic Root** | **WT (N/n=6/10)** | | | |  | **HET (N/n=5/8)** | | | |  |
| --- | --- | --- | --- | --- | --- | --- | --- | --- | --- | --- |
|  | **Median** | **Min** | **Max** | **Mode** |  | **Median** | **Min** | **Max** | **Mode** | ***p***  ***value*** |
| **Intraplaque Macrophages** | 1 | 0 | 2 | 1 |  | 1 | 0 | 2 | 1 | *0.05* |
| **External Macrophages** | 1 | 0 | 3 | 0 |  | 1 | 0 | 2 | 0 | *0.57* |
| **Intraplaque T cells** | 0 | 0 | 1 | 0 |  | 0 | 0 | 1 | 0 | *0.70* |
| **External T cells** | 2 | 0 | 3 | 2 |  | 1 | 0 | 2 | 1 | *0.07* |
